# Bone metastasis as primary presentation of pancreatic ductal adenocarcinoma: A case report and literature review

**DOI:** 10.1002/ccr3.2412

**Published:** 2019-09-05

**Authors:** Antonella Argentiero, Angela Calabrese, Antonio Giovanni Solimando, Antonio Notaristefano, Marzia M.G. Panarelli, Oronzo Brunetti

**Affiliations:** ^1^ Medical Oncology Unit National Cancer Research Centre Istituto Tumori "Giovanni Paolo II" Bari Italy; ^2^ Radiology Unit National Cancer Research Centre Istituto Tumori "Giovanni Paolo II" Bari Italy; ^3^ Department of Biomedical Sciences and Human Oncology Section of Internal Medicine “G. Baccelli” University of Bari Medical School Bari Italy; ^4^ Nuclear Medicine Unit SS Annunziata Hospital, Taranto Bari Italy; ^5^ Division of Anatomic Pathology SS Annunziata Hospital, Taranto Bari Italy

**Keywords:** bone metastases, pancreatic ductal adenocarcinoma, PDAC, zoledronic acid

## Abstract

PDAC bone metastases represent a clinical challenge characterized by multifaceted biological entity.

## 1 INTRODUCTION

Bone metastases are more frequent in prostate, breast, and lung cancers.^1^ Their occurrence represents a clinical issue leading to hypercalcemia, severe pain, and risk of spinal cord injury contributing to an unfavorable prognosis and a much worse quality of life.^2^ Gastrointestinal tumors metastasize less common to the skeleton with a particular low incidence in PDAC patients whose disease is characterized by a more frequent metastatization to liver, peritoneal cavity, and lungs.^3^, ^4^, ^5^, ^6^, ^7^ The most common site of bone metastases is the spine which is associated with back pain that could be confused with symptoms correlated with the primary tumor.^8^ In a few cases, the skeleton may represent its sole metastatic site.^6^, ^9^

In this study, we describe a rare case of bone vertebral metastasis from PDAC which appeared at the onset of the disease.

## 2 CASE PRESENTATION

**Figure 1:**
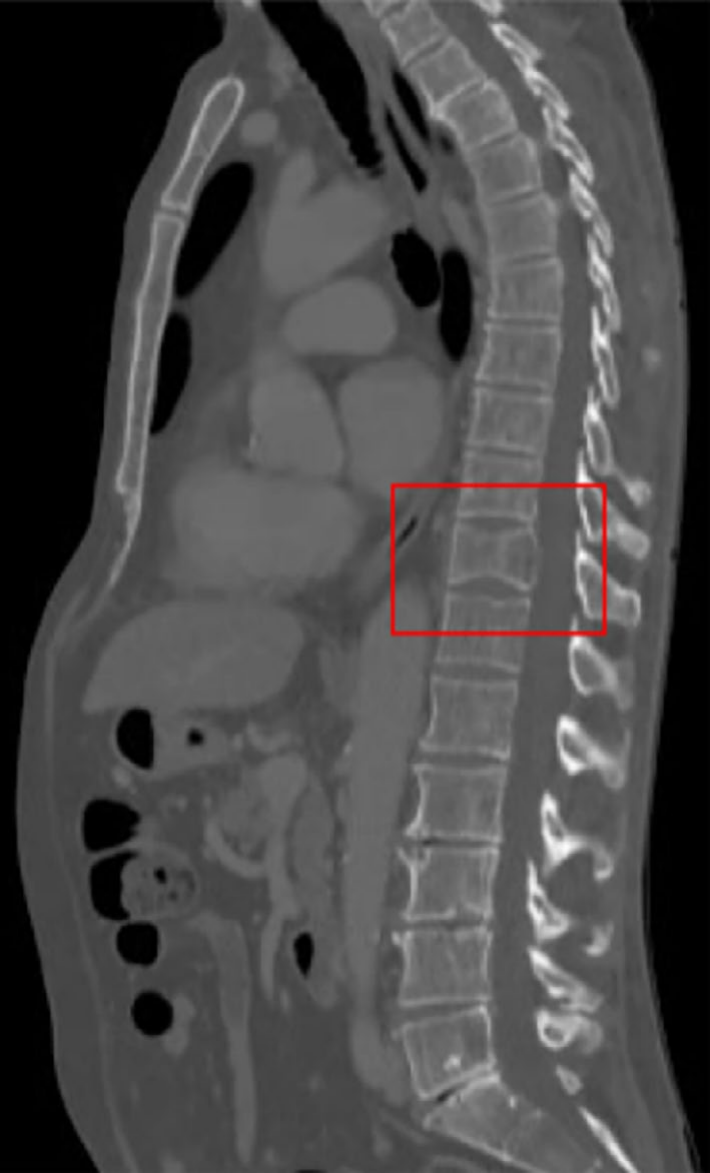
Computerized tomography images of vertebral column revealed bone rarefaction with D11 vertebral fracture and spinal cord compression

A 64‐year‐old man presented severe back pain. He did not report trauma or comorbidities for osteoarticular pathologies in his past medical history. Drugs prescribed for pain control did not relieve symptoms. X‐ray examination showed mild lumbar right‐convex scoliosis and signs of osteophytic spondylosis. Vertebral discomfort and aggravating pain at the level of D11 and D12 vertebrae prompted a neurological assessment which revealed slight inferior limb numbness and weakness, mainly in the left leg in combination with hypoesthesia and dysesthesia in the same region; perineal reflexes were present. Computerized tomography images of vertebral column revealed bone rarefaction with D11 vertebral fracture and spinal cord compression (Figure 1). Laboratory tests showed an increase of carcinoembryonic antigen serum levels (61 ng/mL), while prostate‐specific antigen, beta 2 microglobulin, and CA 19.9 were within the normal ranges.

**Figure 2:**
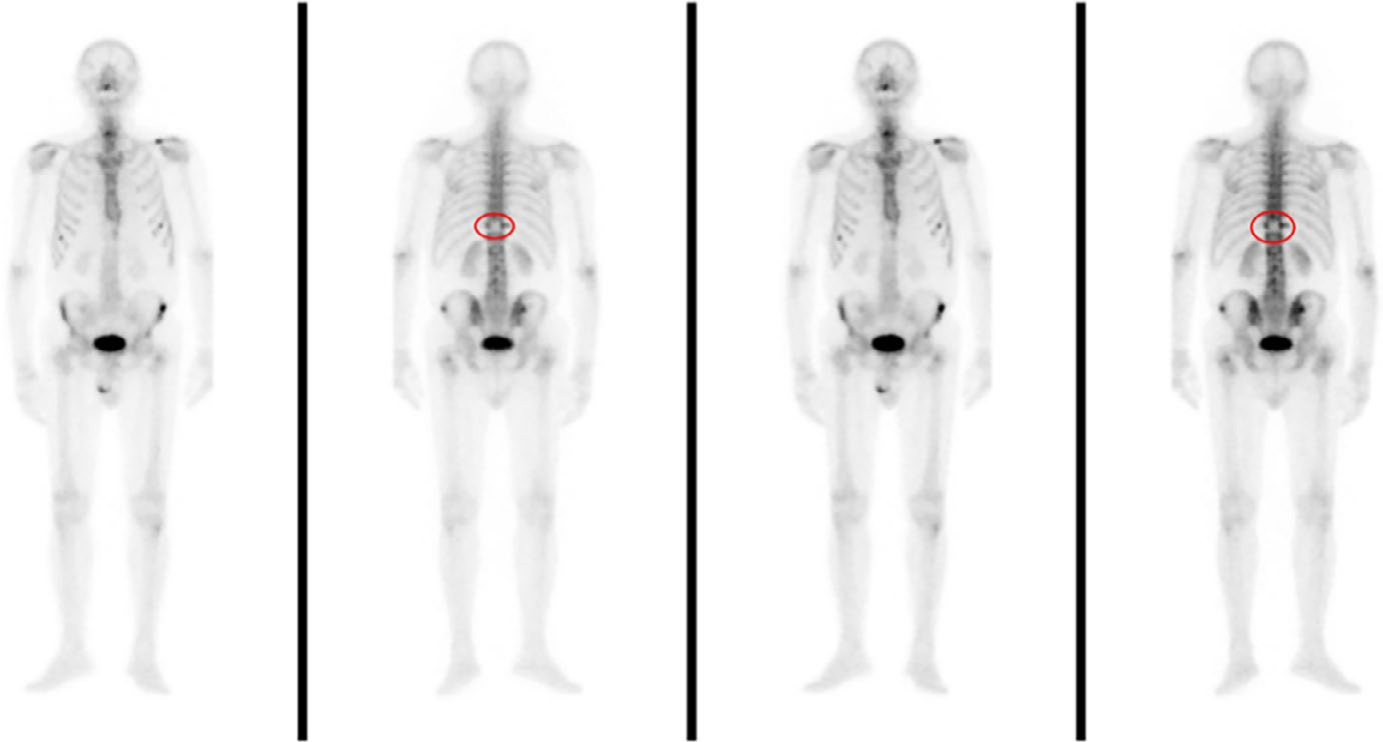
Bone scan showed a hypocaptation area at D11 vertebral, and abnormal uptaking in the right iliac wing, iliac crest, left clavicle, bilateral ribs, and D10 vertebra. In the red rounds the result of percutaneous vertebroplasty

**Figure 3:**
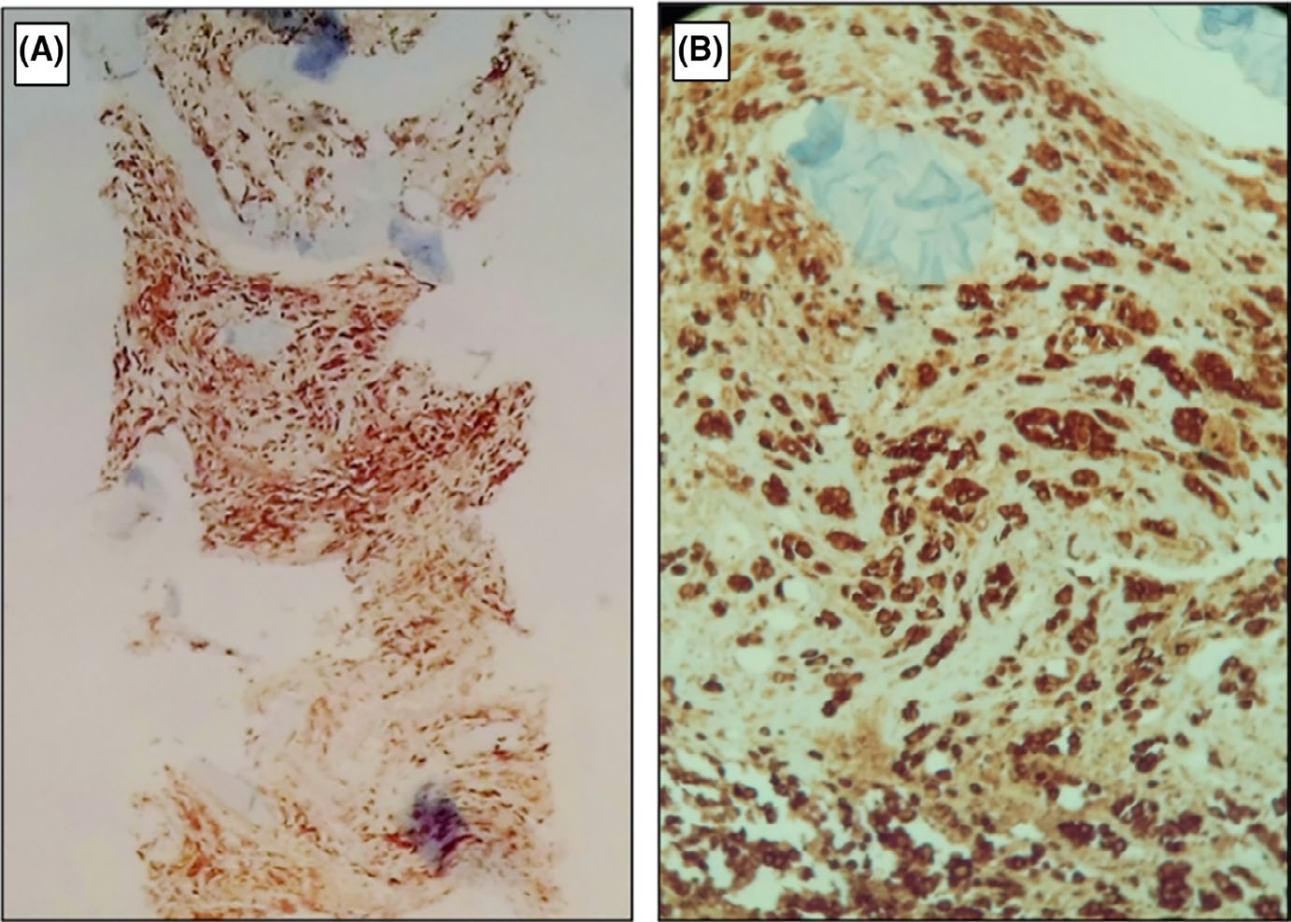
A, Vertebral biopsy speciem 2.5×, CKAE1/AE3 diffusely positive. B, 10×, CKAE1/AE3 diffusely positive in metastasis of adenocarcinoma with acinar pattern

Total‐body computerized tomography revealed only a 23 × 14 mm ill‐defined mass at the pancreatic tail with loss of acinar structure. Percutaneous vertebroplasty in emergency with vertebral biopsy was performed. Bone scan showed a hypocaptation area at D11 vertebral, and abnormal uptake in the right iliac wing, iliac crest, left clavicle, bilateral ribs, and D10 vertebra (Figure 2). Histopathological examination of vertebra soft tissue biopsy led to the diagnosis of metastatic adenocarcinoma with features resembling pancreatic cancer (Figure 3A‐B).

A first‐line chemotherapy regimen was administered with gemcitabine plus nab‐paclitaxel in combination with zoledronic acid. Unfortunately, an early disease bone and liver progression was observed with an overall survival of 6 months.

## 3 DISCUSSION

PDAC is thought to become the second leading cause of cancer death by 2030 with a 5‐year overall survival rate around 7%.^10^, ^11^ The metastatic process is strictly connected to tumor intrinsic and extrinsic characteristics.^12^ As in other several solid and hematologic cancer,^13^, ^14^, ^15^ the tumor microenvironment emerged as a pivotal driver of metastatic niche 16 being correlated at the same time to cell‐adhesion dependent and independent drug‐resistance development.^17^, ^18^, ^19^, ^20^ Thus, several approaches have been envisioned in order to molecularly overcome this malignant phenotype, in order to target both the cancer cells and the tumoral *milieu*.^21^, ^22^, ^23^ Variable incidence of bone metastases from PDAC reported in literature (from 5% to 20%) should be conditioned by either the possible overlapping between symptoms related to the primary tumor and bone localization or the longer survival obtained in the last few years due the availability of new and more active chemotherapy regimens in both adjuvant and advanced settings.^6^, ^24^, ^25^, ^26^, ^27^ The predominance of osteolytic or osteoblastic bone metastasis is controversial in these patients.^8^, ^9^, ^28^

We reported a case of a metastatic PDAC with an osteoblastic/osteolytic bone involvement of both the right iliac wing and the body of the D11 and L3 vertebrae, the main sites of bone colonization in PDAC. Diagnostic biopsy was performed during the vertebroplasty, while the diagnosis of a primitive pancreatic cancer was performed through radiological images.

Our choice of the gemcitabine plus nab‐paclitaxel systemic regimen was supported by an intriguing preclinical study demonstrating that association between nitrogen‐containing bisphosphonates (ie, zoledronic acid) and nab‐paclitaxel reduced fibrosis, peritoneal dissemination, angiogenesis, and cell proliferation.^26^, ^29^ Furthermore, in vitro studies showed an antitumor activity of zoledronic acid against PDAC cells.^30^, ^31^ In addition, zoledronic acid has been reported to stimulate lymphocytes antitumor response through γδ‐type T cell receptors and impact on the median time to the first skeletal‐related event and on overall survival.^32^, ^33^, ^34^, ^35^, ^36^ Remarkably, also innate immunity seems to be related to zoledronic acid efficacy, via tumor‐associated macrophages (TAM).^37^ Nonetheless, we observed a very poor survival in our patient.

Scanty data are available in the literature concerning the clinical outcome of PDAC patients with bone metastases. Borad et al reported a series of seven patients who developed skeletal metastases between 2 and 17.3 months (median: 5.5 months) after the onset of the disease. Survival from the evidence of skeletal metastases ranged between 0.3 and 9 months. Most of these patients (71.4%) received bisphosphonates in combination with systemic chemotherapy.^8^ Rades et al collected data of 15 PDAC with metastatic epidural spinal cord compression from PDAC who underwent radiotherapy. Three of them showed an improvement of motor function, while the others demonstrated no further progression or deterioration. This improvement was significantly associated with the absence of visceral involvement at the time of radiotherapy (*P* = .025), with a 6‐month survival rate of 33%. Moreover, the same authors demonstrated that radiotherapy of 1 × 8 Gy appeared to be not inferior to multi‐fraction radiation schedules considering the post‐treatment motor function in patients with vertebral involvement in PDAC.^38^ Habermehl et al described data of 33 patients with PDAC affected by bone metastases. The investigators showed a median overall survival of 3.1 months (95% CI 1.9‐4.3) and presented survival rates of 75.3%, 46.5%, and 19.9% after 1, 3, and 6 months, respectively. Treatment protocols were different, with most patients treated with 3000 cGy in 10 fractions and a median treatment duration of 15 days.^39^ Few reports described the role of surgery in the management of bone involvement in PDAC. Chih et al reported a patient with L2 vertebra involvement treated with percutaneous vertebroplasty with an improvement of performance status and pain control.^40^

Survival of our patient was lower than the median overall survival of a metastatic PDAC patient treated with first‐line gemcitabine and nab‐paclitaxel. In previous reports, we described a relationship between bone metastases and poorer prognosis in patients affected by gastric cancer and hepatocellular carcinoma.^34^, ^36^ Recently, several data have suggested a potential prognostic and predictive role of molecular characterization in PDAC patients.^41^ As emerged for invasive PDAC the genomic landscape can represent the new frontier to envision therapeutic solutions for aggressive disease.^42^, ^43^

Conclusively, we hypothesize that bone metastatization could represent the phenotypic expression of the underlying molecular PDAC subgroups characterized by unfavorable outcome. This patient's subgroup might be characterized by peculiar prognostic feature, being potentially candidate to specific targeted therapies.^44^

## CONFLICT OF INTEREST

The authors have no conflict of interests.

## AUTHOR CONTRIBUTION

AA, OB, AGS: Conceptualization, AC, AA, AN and AC: methodology, MMGP, AA, AN, AC and AGS: formal analysis, AA, OB and AGS: investigation, AA, MMGP and AN: data curation, AA, OB and AGS: writing—original draft preparation, AA and OB: writing—review and editing, AA and OB: resources, OB and AGS: supervision, AGS: funding acquisition.
